# Development of a Multi-Species Biotic Ligand Model Predicting the Toxicity of Trivalent Chromium to Barley Root Elongation in Solution Culture

**DOI:** 10.1371/journal.pone.0105174

**Published:** 2014-08-13

**Authors:** Ningning Song, Xu Zhong, Bo Li, Jumei Li, Dongpu Wei, Yibing Ma

**Affiliations:** 1 National Soil Fertility and Fertilizer Effects Long-term Monitoring Network/Institute of Agricultural Resources and Regional Planning, Chinese Academy of Agricultural Sciences, Beijing, P.R. China; 2 Institute of Plant Nutrition and Environmental Resources, Liaoning Academy of Agricultural Sciences, Shenyang, P.R. China; University of Nottingham, United Kingdom

## Abstract

Little knowledge is available about the influence of cation competition and metal speciation on trivalent chromium (Cr(III)) toxicity. In the present study, the effects of pH and selected cations on the toxicity of trivalent chromium (Cr(III)) to barley (*Hordeum vulgare*) root elongation were investigated to develop an appropriate biotic ligand model (BLM). Results showed that the toxicity of Cr(III) decreased with increasing activity of Ca^2+^ and Mg^2+^ but not with K^+^ and Na^+^. The effect of pH on Cr(III) toxicity to barley root elongation could be explained by H^+^ competition with Cr^3+^ bound to a biotic ligand (BL) as well as by the concomitant toxicity of CrOH^2+^ in solution culture. Stability constants were obtained for the binding of Cr^3+^, CrOH^2+^, Ca^2+^, Mg^2+^ and H^+^ with binding ligand: log *K_CrBL_* 7.34, log *K_CrOHBL_* 5.35, log *K_CaBL_* 2.64, log *K_MgBL_* 2.98, and log *K_HBL_* 4.74. On the basis of those estimated parameters, a BLM was successfully developed to predict Cr(III) toxicity to barley root elongation as a function of solution characteristics.

## Introduction

Chromium is one of the most widely used metals in modern industry [1], and it could be transferred into the environment through the waste products during various industrial processes [2], [3]. It has, therefore, become a common contaminant in waters and soils. Chromium occurs in the environment primarily in two common oxidation states: trivalent chromium (Cr(III)) and hexavalent chromium (Cr(VI)) [4]. Although Cr (III) is slightly less toxic than Cr (VI), exposure to excess Cr(III) could inhibit plant growth, and in humans it may decrease immune system activity [5]. Over recent years, several researches have been performed to determine the Cr(III) toxicity to plants [5], [6] and terrestrial invertebrates [7], [8]. However, most reported toxicity data were obtained based on total chromium concentration. Little focus on research into the influence of competition and speciation on Cr toxicity. Studies in aquatic toxicology have shown that the competition of other cations and the speciation of metals pose great influence on their toxicity [9], [10]. Therefore, a risk assessment considering the effects of cation competition and metal speciation is needed to properly assess the risk of Cr(III).

A biotic ligand model (BLM) has been developed to predict metal toxicity in aquatic systems [11], which incorporates metal complexation and speciation in the solution surrounding the organisms as well as interactions between metal ions and competing cations at the binding sites on the organism-water interface. The main assumption of the BLM is that metal toxicity is caused by free metal ions reacting with biological binding sites. The cations of H^+^, Ca^2+^, Mg^2+^, Na^+^ and K^+^ might compete with metal ions for these binding sites and decrease the toxicity of the free metal ions [12], [13]. The complexation capacity of the BL and stability constants for the metal-BL and the cation-BL complexes have to be incorporated in a speciation model such as Visual MINTEQ [14] or WHAM (Windermere Humic Aqueous Model) [15] which allows to determine calculation of the free metal ion activity and speciation on basis of the water characteristics. So far the BLMs have been successfully applied in predicting the bioavailability and toxicity of several metals in aquatic systems and partially in terrestrial systems [9], [16], [17]. Thakali et al. [18], [19] have developed a terrestrial BLM and successfully applied it to Cu and Ni toxicity to several biological endpoints (such as barley root elongation) only in noncalcareous soils with pH≤7, because in calcareous soils, the free activity of metals predicted by speciation models has not been solved. Li et al. [9] refined a BLM to predict acute Ni toxicity to barley (*Hordeum vulgare*) root elongation in solution culture, and suggested that Ni^2+^ plus NiHCO_3_^+^ as toxic species and the competition of H^+^, Mg^2+^ and Ca^2+^ with the binding sites of BL should be incorporated in the BLM. The general goal in the study was to develop a Cr(III)-BLM in solution culture, in order to further apply it to predict the toxicity of Cr(III) in aquatic and terrestrial systems.

To our knowledge, no data are available on the effect of pH, competing cations and Cr speciation on Cr(III) toxicity to plants in solution culture and there is no BLM applied to predict acute Cr(III) toxicity to plants. The objectives of the present study were: (1) to investigate the effect of H^+^ competition on the toxicity of Cr(III) to barley root elongation across a wide range of pH values and to determine whether other Cr species are involved in toxicity responses; (2) to determine the effects of Ca^2+^, Mg^2+^, Na^+^ and K^+^ on Cr(III) toxicity to barley root elongation across a wide range of ion concentrations in order to obtain conditional binding constants for Cr^3+^ as well as other cations with BLs; and (3) to establish a multi-species BLM that can be used to predict Cr(III) toxicity to barely for a wide range of solution characteristics.

## Materials and Methods

### Experimental design

To assess the independent effect of different cations on Cr(III) toxicity, the target cation concentrations varied during one-set experiments, while all other cation concentrations were kept low and constant [8], [20]. Five sets of Cr(III) bioassays were performed: Ca-set, Mg-set, Na-set, K-set and pH-set (Table 1). Each set consisted of a series of tested solutions, in which only the concentration of target cation varied, while CaCl_2_ was kept at 0.2 mM as background electrolyte. There were seven concentrations of Cr(III) (as CrCl_3_•6H_2_O) plus one treatment without added Cr^3+^ as a control for all series, and the concentrations of Cr^3+^ in solution ranged from 0 to 25 µM. The concentrations of selected cations were based on the ranges that occur in natural soil pore waters [21].

**Table 1:** Chemical composition of the solution cultures used in the different sets and the measured Cr toxicity threshold at 50% inhibition expressed by total concentration of Cr(III) *(*EC50[Cr_T_]), free Cr activity (EC50{Cr^3+^}) and CrOH^2+^ activity (EC50{CrOH^2+^}) for barely root elongation with 95% confidence intervals.

Sets	Treatments	The values of EC50 and 95% confidence intervals (nM)		
		EC50[Cr_T_]	EC50{Cr^3+^}	EC50{CrOH^2+^}
Ca^2+^ (mM)	0.2	1333(1097–1620)	17.3(14.3–21.1)	1033(850–1256)
	1	2162(1710–2734)	25.0(19.8–31.5)	1489(1181–1877)
	2	3082(2579–3682)	32.7(27.4–39.1)	1951(1631–2335)
	5	4137(3659–4677)	37.7(33.3–42.6)	2246(1986–2540)
	10	5021(4295–5870)	41.6(34.1–50.8)	2480(2027–3033)
	15	6383(5299–7690)	44.9(37.3–53.9)	2674(2203–3219)
Mg^2+^ (mM)	0.05	1249(953–1636)	16.0(12.2–20.9)	952(726–1248)
	0.20	1521(1105–2095)	18.6(13.5–25.7)	1110(805–1530)
	0.50	2020(1491–2737)	23.3(15.9–34.1)	1387(947–2032)
	1.00	2785(2265–3423)	29.6(23.8–36.7)	1761(1420–2187)
	2.00	3367(2614–4337)	32.2(24.0–43.1)	1919(1433–2570)
	4.00	5259(4139–6682)	42.8(35.4–51.7)	2552(2112–3084)
K^+^ (mM)	0.1	1386(1149–1602)	15.8(13.2–18.8)	939(789–1120)
	1.0	1570(1392–1770)	16.4(14.8–18.7)	957(728–1257)
	2.5	1678(1204–2338)	16.2 (11.6–22.5)	993(882–1171)
	5.0	1851(1347–2544)	16.6(12.1–22.8)	991(722–1362)
	7.5	2039(1816–2289)	17.3(15.4–19.4)	1032(919–1159)
	10	2097(1865–2358)	16.9(15.1–19.0)	1011(890–1136)
Na^+^ (mM)	2.5	1194(961–1484)	15.4(12.4–19.2)	921(740–1146)
	5.5	1280(1061–1544)	15.7(13.0–18.9)	932(773–1124)
	10.5	1326(1185–1484)	15.3(13.7–17.1)	911(814–1019)
	15.5	1395(1156–1684)	15.0(12.4–18.1)	893(740–1077)
	20.5	1611(1365–1902)	16.4(13.9–19.3)	977(827–1153)
	25.5	1644(1371–1971)	16.0(13.3–19.2)	953(753–1143)
pH	4.5	537(464–622)	66.8(57.7–77.3)	398(343–462)
	5.0	764(662–881)	32.7(28.2–37.8)	621(538–717)
	5.5	1301(1189–1423)	16.9(15.5–18.5)	1011(922–1109)
	6.0	2457(2096–2880)	7.59(6.47–8.90)	1431(1218–1679)
	6.25	4037(3602–4525)	4.95(4.43–5.95)	1663(1488–1859)

### Solution composition

The chemicals used were all analytical reagent, and deionized water was used during experiments. Tested solution cultures were prepared by adding different volumes of stock solutions of CaCl_2_, MgSO_4_, NaCl and KCl into deionized water. Except for pH-set, these media were adjusted to pH 5.50. For pH-set, the pH values were adjusted to a series of pH from 4.50 to 6.25. The value of pH was controlled using 1 mM MES-buffering (2-[N-morpholino] ethane sulfonic acid) and adding NaOH. MES was chosen because it does not form complexes with Cr (III) [22]. The values of pH and Eh in the nutrient solutions were tested before and after the bioassay using a pH meter (Delta 320, Mettler, Zurich, Switzerland) and a Eh meter (9678BNWP, Thermo, Chelmsford, America). To reach near-equilibrium conditions, media were prepared one day before the start of the bioassay. For all treatments, the pH values decreased by 0.05–0.20 (pH unit) when compared with the initial pH. The Eh values of different treatments were various, ranging from 328 to 452 mV. The chemical characteristics of the different tested solution cultures are summarized in Table 1.

### Toxicity assays

The barely root elongation test was performed according to ISO guideline 11269-1 (ISO, 1993). Barley (*H. vulgare*) seeds were surface-sterilized in 2% NaClO for 30 min, after which they were thoroughly rinsed with deionized water and germinated on filter paper moistened with demonized water for 36 h at 20°C in the dark. When the radical emerged (approximately 2 mm in length), six seedlings were transferred to nylon net fixed on the surface of polypropylene pots containing 250 mL exposure solutions. There were three replicate pots for each exposure concentration. The culture containers were placed randomly in a growth chamber. The air temperature was maintained at 20°C during the 16 h (22 k lux)/8 h dark cycles. Root length was measured after 5 d and elongation (RE, %) was calculated as percentage of the control using the equation as follows:
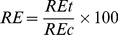
(1)where *RE*t is the root length in the tested solution culture and *RE*c is the root length in the control.

### Chemical measurements

Atomic absorption spectrophotometry (Varian AA240FS/GTA120; Melbourne, Australia) was used to determine the concentration of Cu, Ca, Mg, Na and K.

The selective exchange resin Dowex-M4195 was used to evaluate whether Cr(III) was oxidized to Cr(VI) in the tested solution during the experiment period according to [23]. The Dowex-M4195 resin (particle size = 40 mesh) was immersed in deionized water for 2 days and washed with 1 M HCl. The resin was saturated with 500 mg L^−1^ CuCl_2_ to reach Cu-saturated state in a glass column. The Cu-saturated resin was washed using deionized water until the effluent Cu concentration could no longer be detected. The Dowex-M4195 resin was transferred into separate flasks with the tested medium and shaken at 25°C for 24 h, and then washed with deionized water and placed into 100 mL of 10% NaCl to desorb the Cr adsorbed on resins until the effluent Cr concentration could no longer be detected. The Cr concentration in the desorbed solution was determined by inductively coupled plasma mass spectrometry (ICP-MS: 7500a, Agilent, Arcade, NY, USA).

### Speciation of Cr in solutions

Speciation was calculated by Visual MINTEQ 3.0 (available at http://hem.bredband.net/b108693/). Input data for Visual MINTEQ were pH and the concentrations of Cr, Ca, Mg, K, Na, Cl and SO_4_. As the experiments were carried out in an open system, a CO_2_ partial pressure of 3.5×10^−4^ atm (1 atm = 101.3 kPa) was assumed in the calculation of Visual MINTEQ.

### Mathematical description of the BLM

Based on the BLM assumption, when the competing cations H^+^, Ca^2+^, Mg^2+^, K^+^ and Na^+^ are considered, the fraction (*f*) of the total biotic ligand sites bound by Cr^3+^ is given by the following equation [7]:
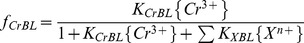
(2)where *K_CrBL_* and *K_XBL_* are conditional binding constants for the binding of Cr and cation X (e.g., Ca^2+^, Mg^2+^, K^+^ or H^+^) to the BL sites (M), respectively, and curly brackets {} indicate ion activity, such as{X^n+^}, which is the activity of X^n+^ (M). {XBL} is the concentration of the specific cation-BL complex (M).

According to the methodology described in detail by De Schamphelaere and Janssen [13], when inhibition of barley root elongation is up to 50% of the control, Eq. (2) becomes:
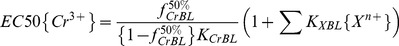
(3)where EC50{Cr^3+^} is the free Cr^3+^ that results in 50% RE (50% of barley root elongation with respect to the control) and 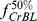 is the fraction of the BLs that results in 50% RE when occupied by Cr. The barley root elongation is correlated to the fraction of the BLs (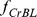) and follows the log-logistic dose-response relationship according to Thakali et al. [18], [19].
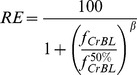
(4)where *β* is the shape parameter. Substituting *f* from Eq. (2) in Eq. (4) yields:



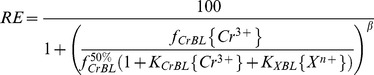
(5)Eq. (5) provides the mathematical basis for the BLM that explicitly relates the biological response to the chemistry of the solution. Meanwhile, the free ion activity model (FIAM) is also fitted to the same dataset as the following equation for comparison with the BLM:
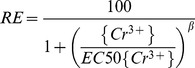
(6)


The dose-response curves are plotted in terms of free Cr^3+^ activity (FIAM) and the fraction (*f*) of the barley root sites bound by toxic Cr species (BLM) by fitting a logistic model. The fitting parameters are conditional binding constants of all cations to BL (*K_MBL_*), 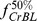 and *β* for BLM and EC50{Cr^3+^} and *β* for FIAM. When comparing different models, the lower value of the root-mean-square error (RMSE) is used as an indicator of the better model:
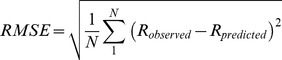
(7)where *N* is the number of data, *R_observed_* the measured *RE* (as % of control) and *R_predicted_* the predicted *RE* (as % of control). The parameters of models were acquired by the mathematic model program in the DPS 9.5 statistical software [24].

## Results

### Distribution of chromium species in different pHs

**Figure 1:**
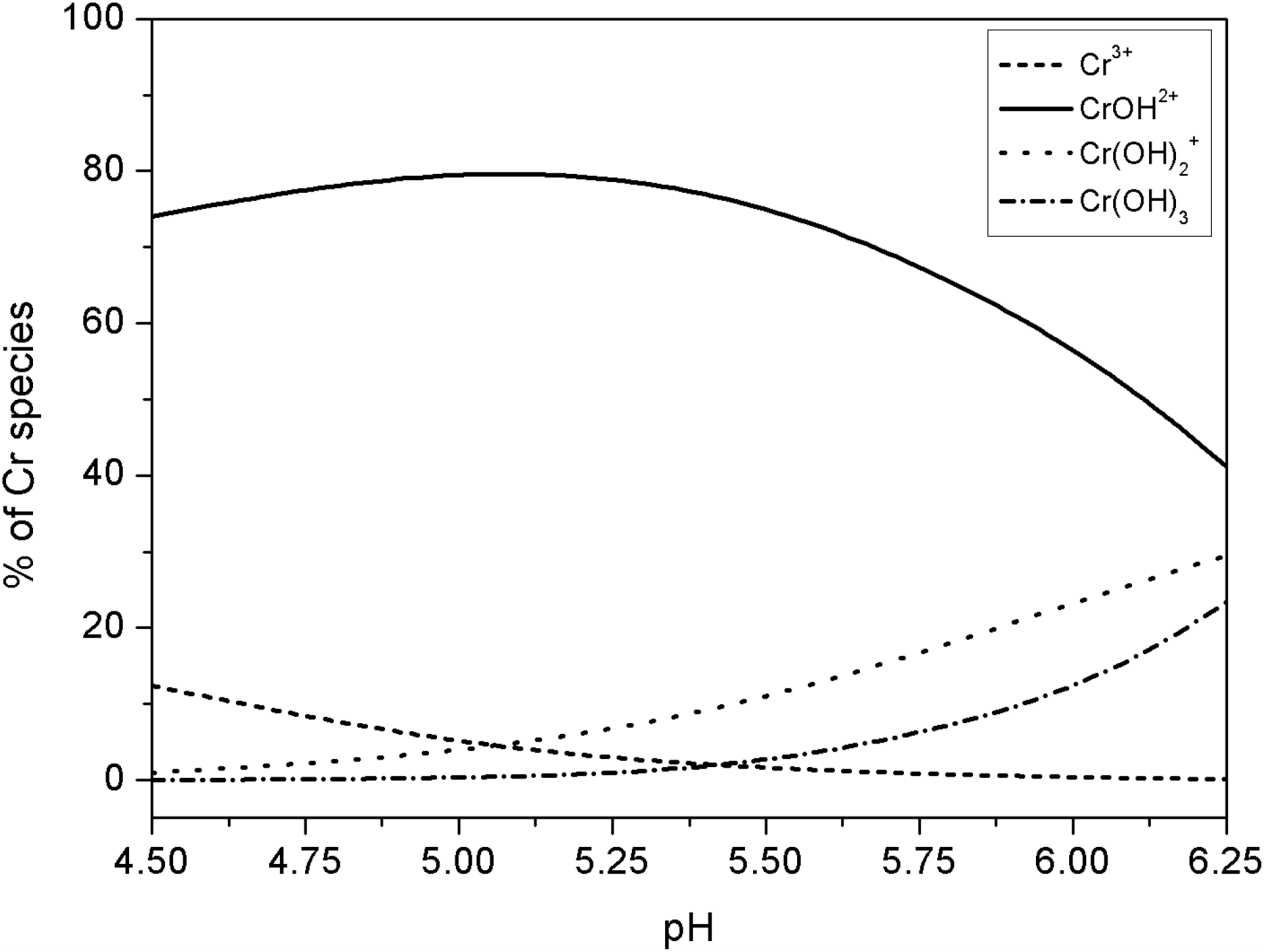
The distribution of different Cr(III) species (%) as a function of pH calculated by Visual MINTEQ.

Resin-extractable Cr by Dowex M4195 was not detected in the test medium, which implied that there was no Cr(III) oxidized to Cr(VI) during the experiment period. The distribution of Cr species in the solutions with pH from 4.5 to 6.25 is shown in Fig. 1. Free Cr^3+^ and CrOH^2+^ were major species at pH 4.5, which were 12.4% and 74.1% of the total Cr, respectively. With increasing pH, the proportion of CrOH^2+^ and Cr^3+^ in solution decreased sharply continuously concomitant with the increasing proportion of Cr(OH)_2_^+^ and Cr(OH)_3_ (aq). At solution pH 6.25, the proportions of Cr(OH)_2_^+^ and Cr(OH)_3_ (aq) reached 29.5% and 23.4% of total Cr, respectively. Other Cr species, such as CrCl^2+^ were always quite low (<0.2% of total Cr) and were not considered for BLM development. Hence, the four main Cr species, Cr^3+^, CrOH^2+^, Cr(OH)_2_^+^ and Cr(OH)_3_ (aq) were considered to test their effects on the toxicity to barley root elongation.

### Effects of cations on Cr toxicity

**Figure 2:**
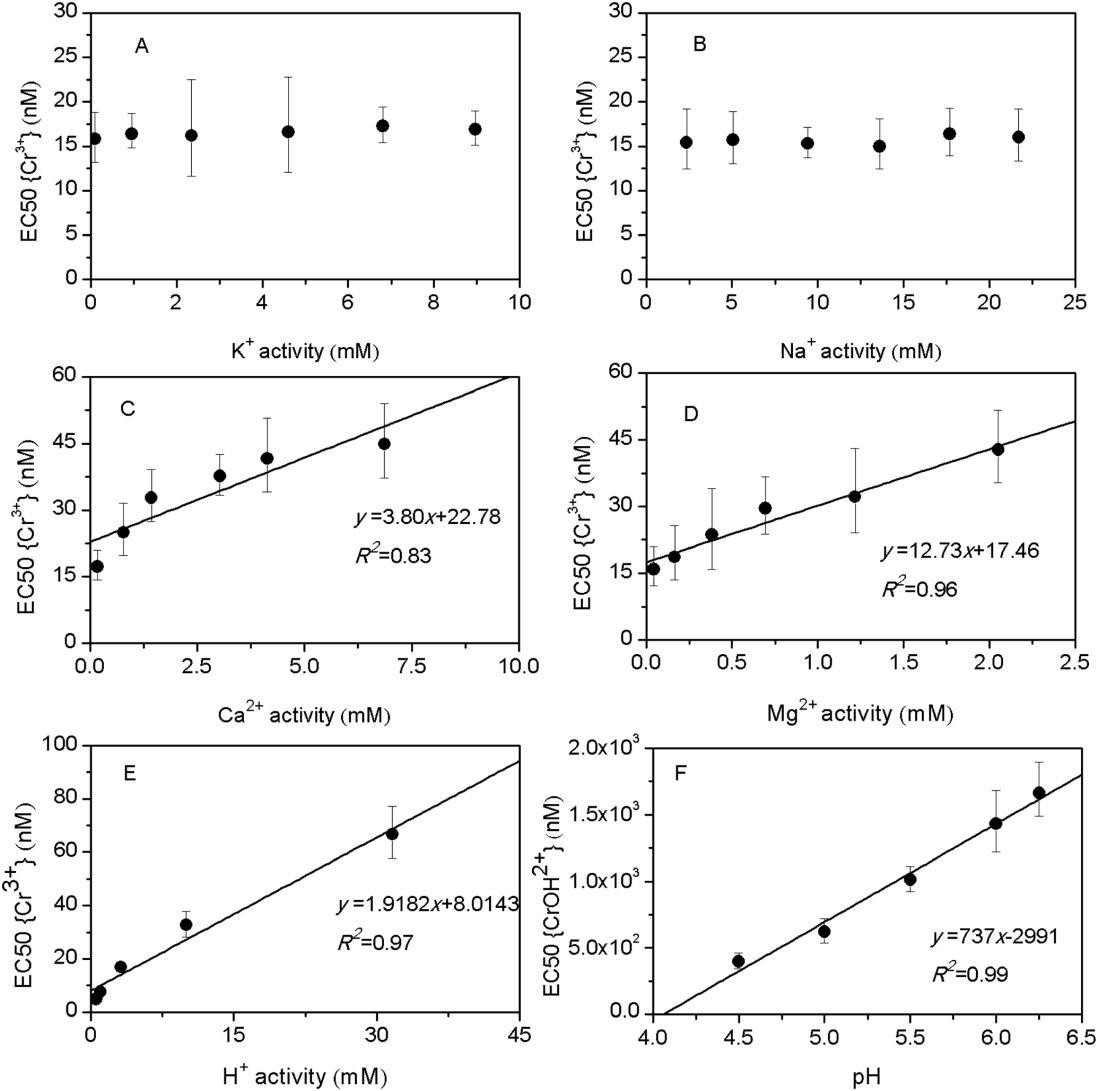
The EC50 values expressed as free Cr^3+^ activity (EC50{Cr^3+^}) for (A, B, C, D, E) or free CrOH^2+^ activity (EC50{CrOH^2+^}) (F) for barley root elongation as a function of the free activity of K^+^ (A), Na^+^ (B), Ca^2+^ (C), Mg^2+^ (D), H^+^ (E), and pH (F). Error bars indicate 95% confidence intervals. Solid lines represent significant correlations.

The EC50 for barley root elongation expressed as free Cr^3+^ activity, ranged from 4.95 to 66.8 nM (Table 1). The values of EC50{Cr^3+^} increased linearly up to 2.59-fold with an increase of Ca^2+^ activity from 0.18 to 6.87 mM (*p*<0.05, *R^2^* = 0.83, Fig. 2C and Table 1). The increase of Mg^2+^ activity from 0.04 to 2.05 mM resulted in the increase of EC50{Cr^3+^} by a factor of 2.68. A linear relationship (*p<*0.01, *R^2^* = 0.96) was found between Mg^2+^ activity and EC50{Cr^3+^} (Fig. 2D and Table 1). However, no significant change in the EC50{Cr^3+^} was found when the activity varied from 0.10 to 8.97 for K^+^, and from 2.35 to 21.7 mM for Na^+^ (Table 1). Therefore, competition between K^+^ and Na^+^ with Cr^3+^ for binding sites on barley roots could be neglected when BLM was developed, and the values of log*K_KBL_* and log*K_NaBL_* could be approximately set to zero.

According to Eq. (3), if H^+^ can compete with Cr^3+^ binding sites of barley root, then a linear relationship between EC50{Cr^3+^} and H^+^ activity should exist in the pH-set. In the present study, the values of EC50{Cr^3+^} increased significantly with an increase of H^+^ activity in culture solution at *p*<0.01 level with *R^2^* = 0.97 (Fig. 2C), which could be explained by H^+^ competition with Cr^3+^ for binding sites of barley root. In addition, from Cr species distribution, it was known that increasing pH from 4.50 to 6.25 resulted in an obvious decrease in the percentage of CrOH^2+^ and an increase in the percentages of Cr(OH)_2_^+^ and Cr(OH)_3_ (aq) to total Cr in solution. To determine whether CrOH^2+^, Cr(OH)_2_^+^ and Cr(OH)_3_ (aq) were toxic to barley root elongation, Eq. (3) was transformed to Eq. (8) when CrOH^2+^, Cr(OH)_2_^+^ and Cr(OH)_3_ (aq) were considered as toxic species as well as Cr^3+^ in the pH set:
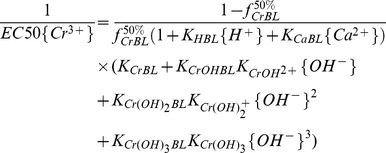
(8)where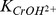, 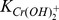
and 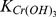
are stability constants for the formation of the CrOH^2+^, Cr(OH)_2_^+^ and Cr(OH)_3_ complexes, respectively. Based on equilibrium equations of Cr^3+^ + OH^−^ = CrOH^2+^, Cr^3+^ +2OH^−^ = Cr(OH)_2_^+^ and Cr^3+^ +3OH^−^ = Cr(OH)_3_, Eq. (8) could be transformed to Eq. (9):



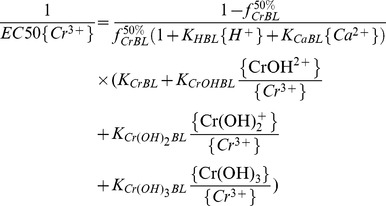
(9)The values of *K_HBL_* and *K_CaBL_* were set to 10^4.74^ and 10^2.64^ in the present study, respectively (see Table 2), and Eq. (9) can be transformed to a equation with 1/EC50{Cr^3+^} as a dependent variable, and 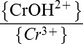 as independent variables. The multiple regression between 1/EC50{Cr^3+^} and *K_HBL_*{H^+^}, CrOH^2+^/Cr^3+^, Cr(OH)_2_^+^/Cr^3+^ as well as Cr(OH)_3_/Cr^3+^ was calculated as:

**Table 2:** Model fit summary and the optimum parameters (±standard errors) associated with the FIAM considering free Cr^3+^ activity only and BLM considering Cr^3+^ plus CrOH^2+^ as toxic species and the competition of H^+^, Mg^2+^ and Ca^2+^for Cr(III) toxicity to barley root elongation.

Model	LogK					*R^2^*	*RMSE*	*EC50*{Cr^3+^}(nM) or 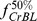	*β*
	Cr^3+^	CrOH^2+^	Ca^2+^	Mg^2+^	H^+^				
FIAM						0.91	8.82	20.15±0.84	0.77±0.03
BLM	7.43±0.11	5.61±0.06	2.64±0.07	2.98±0.08	4.74±0.19	0.97	5.15	0.38±0.06	1.50±0.04

The parameters were estimated using the DPS 9.5 statistic software. *RMSE* represents the root-mean-squared error of the predicted % root elongation. *R^2^* was the determination coefficient of the models between the measured and the predicted % root elongation).



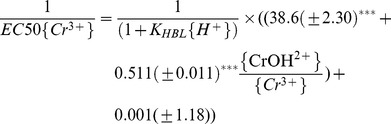
.
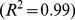
(10)


According to Eq. (10), it was indicated that both the intercept and the coefficient of CrOH^2+^/Cr^3+^ were significant at *p*<0.001 level, which demonstrated that Cr toxicity to barely root elongation could be caused by Cr^3+^ plus CrOH^2+^ when they exist in solution at certain pH values. These data suggested that the effects of Cr(OH)_2_^+^ and Cr(OH)_3_ on the total Cr toxicity at pH 4.50–6.25 could be ignored and the toxicity of Cr^3+^ plus CrOH^2+^ should be considered in the Cr(III)-BLM development.

### Estimation of BLM parameters

When the toxicity of CrOH^2+^ was considered, Eq. (4) can be transformed to Eq. (11) [13]:
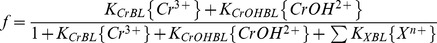
(11)


Then barley root elongation could be written as follows:
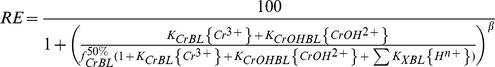
(12)


From Eq. (12), barley root elongation was affected by {Cr^3+^}, {CrOH^2+^}, {H^+^}, {Mg^2+^} and {Ca^2+^}, where {Na^+^} and {K^+^} were expelled from Eq. (12) because their effects on Cr toxicity were insignificant in the present study. So, Eq. (12) can be written as:
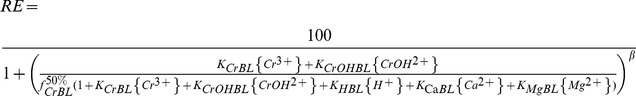
(13)


**Figure 3:**
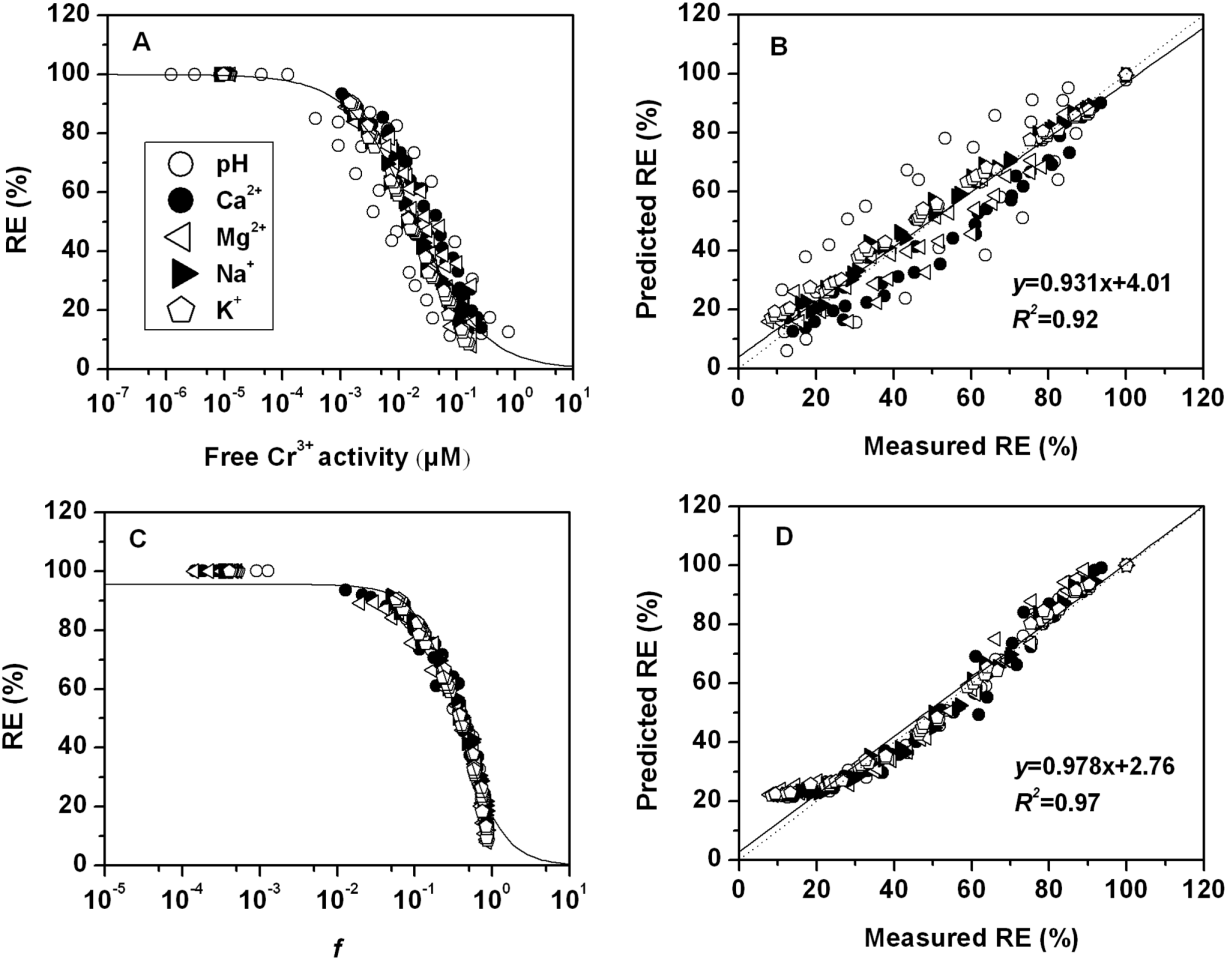
Toxicity of Cr to barley root elongation expressed as different dose–response curves: the dose as free Cr^3+^ activity only (A), as the fraction (*f*) of the total biotic ligand sites occupied by toxic Cr^3+^ and CrOH^2+^ considering the competitive effect of Ca^2+^ and Mg^2+^ activity (C). The measured versus predicted root elongation based on FIAM(B) and BLM(D).The dotted lines are 1∶1 lines and the solid lines represent the linear regression relationships between the measured and predicted barley root elongation. The lines are the fitted logistic curves based on all sets.

The parameters, *K_CrBL_*, *K_CrOHBL_*, *K_HBL_*, *K_CaBL_*, *K_MgBL_*, 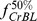 and *β* can be obtained by data fitting the predicted *RE* (% of control) with minimal *RMSE* and maximal *R^2^* for all sets using the DPS 9.5 statistic software. The conditional binding constants were obtained as follows: log*K_CrBL_* = 7.43, log*K_CrOHBL_* = 5.61, log*K_HBL_* = 4.74, log*K_CaBL_* = 2.64 and log*K_MgBL_* = 2.98 (Table 2). Those results indicate that toxicity across the wide range of pH and concentration of cations is closely related to activities of Cr^3+^ and CrOH^2+^ as well as competition with H^+^, Mg^2+^ and Ca^2+^ to barley root binding sites, which should be incorporated in the Cr(III)-BLM. Therefore the dose–response curves were plotted in terms of free Cr^3+^ activity based on FIAM, and in terms of *f* (fraction of the total barley root sites occupied by toxic Cr^3+^ and CrOH^2+^ species) with considering the competitive effect of H^+^, Ca^2+^ and Mg^2+^ based on the BLM (Fig. 3). Based on *RMSE* and *R^2^* values, the BLM considering the metal speciation and competing cations was able to predict Cr toxicity better than the FIAM. The *RMSE* decreased from 8.82 for FIAM to 5.15 for BLM, and the *R^2^* value increased from 0.91 for the FIAM to 0.97 for the BLM. Also, considering the influence of Cr^3+^, CrOH^2+^, H^+^, Ca^2+^ and Mg^2+^, the BLM clearly showed the best fit with the measured versus predicted values with intercept nearest 0 and the slope nearest 1 (Fig. 3D). The results indicate that BLM can predict barley root elongation much better than FIAM when Cr^3+^ plus CrOH^2+^ as toxic species and the competition of H^+^, Mg^2+^ and Ca^2+^ with the binding sites of barley root are incorporated in the Cr(III)-BLM.

### Validation of BLM

In attempt to examine the prediction ability of the developed Cr(III)-BLM for barley root elongation, an auto-validation was performed based on measured and predicted EC50{Cr^3+^}. The predicted equation of EC50{Cr^3+^} can be expressed as follows based on Eq. (3):
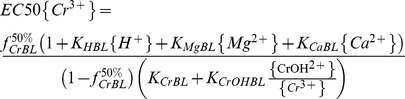
(14)


**Figure 4:**
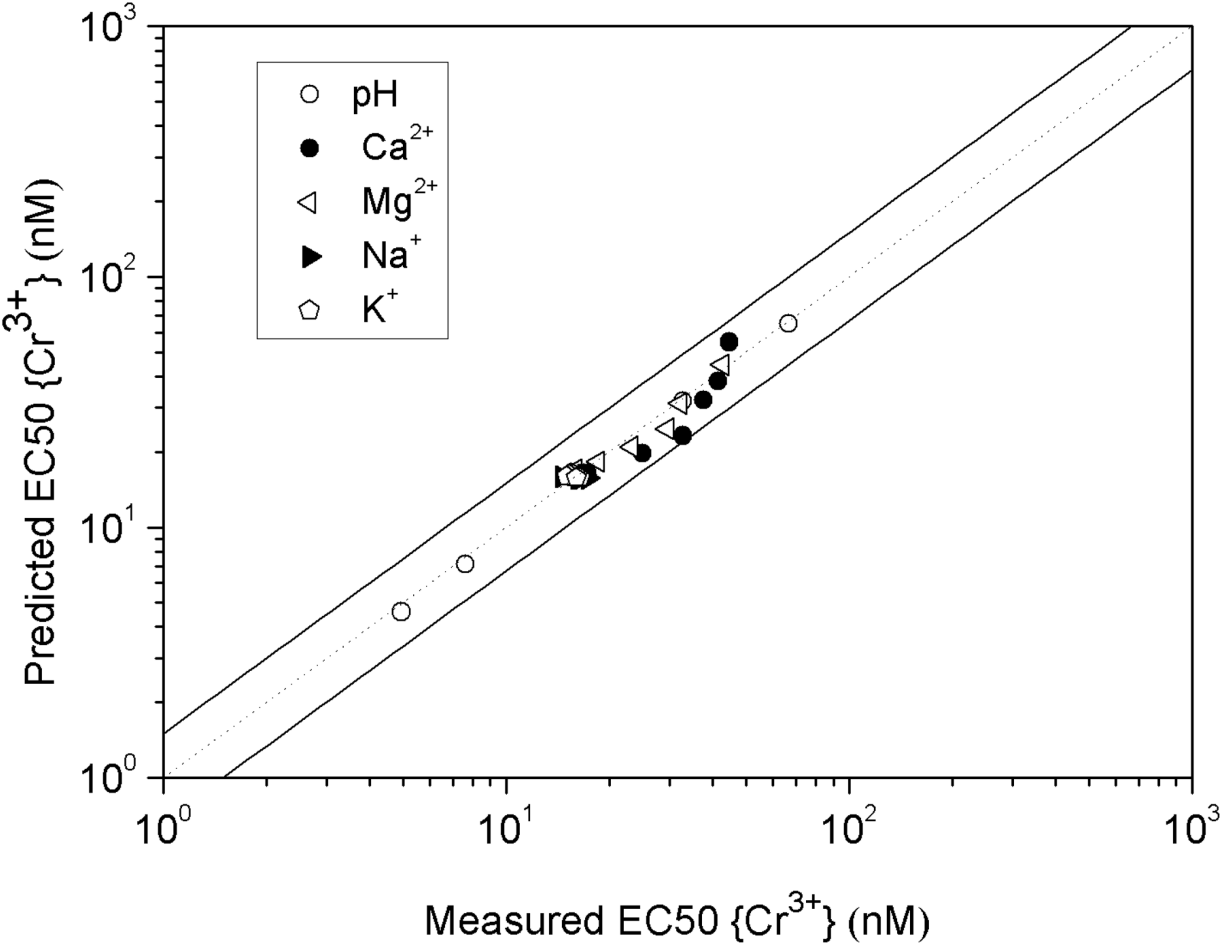
Relationship between the measured and predicted EC50{Cr^3+^} based on the BLM developed in the present study. The solid line indicates a perfect match between measured and predicted EC50{Cr^3+^} values, and the dashed lines indicate the range of a factor of 1.5 between observed and predicted EC50{Cr^3+^} values.

Na^+^ and K^+^ were excluded from the EC50{Cr^3+^} prediction due to their insignificant effects on EC50{Cr^3+^} values of barley root elongation. The corresponding parameters (*K_HBL_*, *K_MgBL_*, *K_CaBL_*, *K_CrBL_*, *K_CrOHBL_* and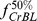) are listed in Table 2. EC50{Cr^3+^} can be predicted when the activities of {H^+^}, {Mg^2+^}, {Ca^2+^}, {CrOH^2+^} and{Cr^3+^} are obtained from Visual MINTEQ. Results from Fig. 4 showed that the predicted EC50s differed from the measured EC50s by less than a factor of 1.5 in the present study, indicating that the BLM can be used to predict Cr(III) toxicity to barley root elongation.

## Discussion

In the present study, the Cr(III) toxicity threshold at 50% inhibition expressed by total concentration of Cr(III), i.e. EC50[Cr_T_], seemed to increase with increasing of K^+^ or Na^+^ activity. However, when the Cr toxicity threshold at 50% inhibition expressed by the activity of free Cr^3+^, i.e. EC50{Cr^3+^}, there was no significant effects of the activity of K^+^ or Na^+^ on Cr(III) toxicity (Table 1 and Fig. 2). The results suggested that the effects of K^+^ and Na^+^ activity on Cr(III) toxicity was attributed to the electrolyte-induced decreases of Cr(III) activity and not competition with Cr(III) for binding sites in *H. vulgare*. Protective effects of Ca^2+^, Mg^2+^ and H^+^ on Cr(III) toxicity to barley were found and the stability constants were derived (Table 2). Many researchers have reported protective effects of major cations and proton (i.e., Ca^2+^, Mg^2+^, K^+^, and H^+^) on the toxicity of several heavy metals [18], [19], [25]. For Cu toxicity, Kinraide et al. [26] reported that Ca^2+^ and Mg^2+^ had a protective effect against Cu toxicity to wheat (*Triticum aestivum*), while Le et al. [27] found that only H^+^ could decrease Cu^2+^ toxicity to lettuce (*Lactuca sativa*) root elongation bioassay significantly. For Ni toxicity, Li et al. [9] found that EC50{Ni^2+^} was correlated significantly with the activity of Mg^2+^, Ca^2+^and H^+^, not with the activity of Na^+^ and K^+^. In the case of Zn toxicity, it appeared that the increase of Mg^2+^ and K^+^ activity could alleviate Zn toxicity to wheat (*T. aestivum*) and radish (*Raphanus sativus*) [28]. The protective effects of Mg^2+^, Ca^2+^, K^+^ and H^+^ on Zn^2+^ toxicity to barley were also found by Wang et al. [29]. The alleviating effects of cations such as Ca^2+^, Mg^2+^, K^+^ and H^+^ on metal toxicity can also be interpreted in terms of membrane-surface electrical potentials [30], [31]. Cell surfaces are negatively charged and these charges create negative potentials at the cell membrane surfaces. Changes in this surface electrical potential may influence the surface activities of free ions and the electrical driving force for ions and hence affect ion transport. Cations such as Ca^2+^ and Mg^2+^ depolarized the plasma membrane and reduced the negativity of the electrical potential at the outer surface of the plasma membrane and thereby alleviate uptake and effects of toxic metals [30].

The relative affinity of the BL sites for the cations, H^+^ > Mg^2+^ > Ca^2+^, was the same order as the results of an acute Ni-BLM for root elongation of *H. vulgare* developed by Li et al. [9] and an acute Zn-BLM for root elongation of *H. vulgare* developed by Wang et al. [28]. The binding constants log *K_HBL_* (4.74) in the present study was found to be lower than that (log *K_HBL_* = 6.48) reported by Thakali et al. [18], [19] in a terrestrial BLM for Cu toxicity to barley root elongation bioassay in soil solutions, whereas it was higher than (log *K_HBL_* = 4.29) reported by Li et al. [9] in the BLM for acute Ni toxicity to barley root elongation and that (log *K_HBL_* = 4.27) reported by Wang et al. [28] in a BLM for acute Zn toxicity to barley root elongation in culture solutions. The value of log *K_CaBL_* (2.64) in the present study was similar with the result of acute Cu-BLM for root growth of *T. aestivum* developed by Luo et al. [29] (log *K_CaBL_* = 2.43), but higher than that (log *K_CaBL_* = 1.60) reported by Wang et al. [28]. The value of log *K_MgBL_* (2.98) in the present study was similar with the result of acute Cu-BLM for root growth of *T. aestivum* developed by Wang et al. [20] (log *K_MgBL_* = 2.92), whereas it was lower than that (log *K_MgBL_* = 4.01) reported by Li et al. [9] and that (log *K_MgBL_* = 3.72) reported by Wang et al. [28]. It was noted that the derived stability constants should be regarded as parameters that reflect the observed relations between the activity of Ca^2+^, Mg^2+^ and H^+^ and the toxicity of metals. Differences in binding constants may, for example, result from different exposure duration, endpoint, target tissue or BL, or mechanisms of the toxicity of metals [8], [20]. More researches with chromium need to be done to investigate the differences and similarities across organisms, endpoints and exposure duration.

The effect of solution pH on the metal activity can be explained, in part, by the competition of H^+^ and other heavy metal ions for the common binding sites, since the pH affects either the solubility and/or the speciation of many metal ions [32]. It has been indicated that besides free metal ions, the inorganic species of metals such as CuOH^+^, ZnOH^+^ and NiHCO_3_^+^ were found also to be toxic to biota in the developed BLMs [9], [12], [13]. Heijerick et al. [33] observed an increase of the acute Zn toxicity to water flea *Daphnia magna* when effective concentrations were expressed as dissolved Zn but not as free Zn^2+^ activity and suggested that the effect of pH on acute Zn toxicity was a speciation effect. Li et al. [9] found that higher H^+^ activity decreased the Ni toxicity to barely through H^+^ competition with Ni^2+^ bound to biotic ligands at pH<7.0 or through the change of Ni species in solution at pH≥7.0, and also Ni^2+^ plus NiHCO_3_^+^ were toxic to barley root elongation in solution at pH≥7.0. Wang et al. [20] studied the acute Cu toxicity to barley root elongation in the pH range 5.98–7.92 and found that the relation between H^+^ and EC50{Cu^2+^} should rather be explained in terms of toxicity of Cu^2+^, plus CuHCO_3_^+^, CuCO_3_ (aq) and CuOH^+^ than in terms of proton competition. In the present study, there was a linear relationship between H^+^ activity and Cr^3+^ toxicity over the whole pH range, and the values of EC50{CrOH^2+^} ranged from 398 to 1663 nM with the increasing pH from 4.50 to 6.25, indicating that the effect of pH on Cr metal toxicity was a significant competition effect as well as speciation effect between protons and metal ions. This finding was consistent with that reported by Cremazy et al. [34], who studied the uptake of a trivalent ion scandium (Sc) by *Chlamydomonas reinhardtii*, and found H^+^ competitive for binding with Sc^3+^ transport sites within the pH range of 4.50 to 6.00, and also suggested that reasonable fit for BLM could also be obtained as a function of the first hydroxo-species ([ScOH^2+^]) along with proton competition. The results from Table 2 showed that Cr^3+^ had a higher affinity to the biotic ligand than CrOH^2+^, which can be correlated to the charge of the ion. It was in agreement with that of Yun et al. [35], who investigated biosorption of Cr(III) using protonated brown algae, *Ecklonia* biomass, and found chromium ions (Cr^3+^ and CrOH^2+^) binding was attributed to carboxylic groups, with values of 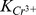 > 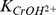
for the biosorption of Cr(III). Based on chemical complexation theory, the affinity of Cr^3+^ for ligands was much higher than CrOH^2+^ which may result in Cr^3+^ being easier to bind to ligands with higher binding constant. In a study of Cr(III) biosorption onto protonated brown algae *Pelvetia canaliculata*, Vilar et al. [36] reported the modeling information on equilibrium and kinetics using the Cr(III) speciation in solution, and found that CrOH^2+^ binding always remained lower than Cr^3+^ and diffused slower than Cr^3+^ even for pH values higher than 3.55, where the concentrations of ions was higher than Cr^3+^ ions. Although Cr^3+^ ions is not the dominated specie of the total Cr(III) at pH 4.50–6.25 (Fig. 1) in the present study, it was expected as one of the dominant toxic forms as well as CrOH^2+^, since it has a higher affinity than CrOH^2+^ to the binding sites.

The 5 d EC50{Cr^3+^} for barely root elongation ranged from 4.95 to 66.8 nM for all treatments and varied about 13-fold, which clearly demonstrates the limitations of using free ion activity for predicting the toxicity of Cr(III). The BLM developed in this study could predict EC50s accurately (difference of factor of 1.5), indicating that it can be used to predict toxicity of Cr(III) to terrestrial plants. However, the application of this Cr(III)-BLM is hampered by the problematic of measuring or predicting metal speciation for the complex mixtures of organic matter in natural soil solutions. Also, when the constants derived in the present study are used to predict Cr(III) toxicity in soil by this Cr(III)-BLM, they still need be validated or further study by the experiments with dissolved organic matter (DOM) additions and with natural soils. The direct links between chemistry of metals in soils and their ecotoxicity might be a good approach in the future [18], [19].

## Conclusions

In the present study, a BLM was developed for predicting the toxicity of Cr(III) to barley (*H. vulgare*) in nutrient solutions. It was found that Cr^3+^ plus CrOH^2+^ as toxic species and competition with H^+^, Mg^2+^ and Ca^2+^ for the binding sites of BL should be incorporated into the BLM. The BLM parameters were derived and validated, and the developed BLM demonstrated good performance in predicting acute Cr(III) toxicity to barley root elongation. The BLM, therefore, may initiate a promising tool for improving the ecological relevancy of risk assessment procedures for trivalent metals such as Cr(III) as well as divalent metals in water and soils.
